# Surgical Treatment of Cervical Spondylotic Myelopathy Associated Hypertension—A Retrospective Study of 309 Patients

**DOI:** 10.1371/journal.pone.0133828

**Published:** 2015-07-20

**Authors:** Ze-qun Li, Yun-peng Zhao, Wen-yu Jia, Xia Wang, Bin Chen, Muhammad Shahbaz, Lin Nie, Lei Cheng

**Affiliations:** 1 Department of Spine Surgery, Qilu Hospital of Shandong University, Jinan, Shandong Province, P. R. China; 2 Department of General Surgery, Qilu Hospital of Shandong University, Jinan, Shandong Province, P. R. China; 3 Department of Endocrinology and Metabolism, Shandong Provincial Hospital affiliated to Shandong University, Jinan, Shandong Province, P. R. China; 4 Institute of Endocrinology, Shandong Academy of Clinical Medicine, Jinan, Shandong Province, P. R. China; Nagoya University, JAPAN

## Abstract

Hypertension is the most prevalent cardiovascular disease, and various risk factors are known to be involved in it. Cervical spondylotic myelopathy (CSM) is the most common non-traumatic cause of myelopathy, which displays neurological symptoms and may induce systemic symptoms. To date, it is still unknown whether CSM is associated with hypertension, and if so, whether the decompression operations can attenuate CSM associated hypertension. Here, a total of 309 patients with CSM who received anterior or posterior decompression surgery were enrolled as subjects. Blood pressure measurements were performed before and within one week after the surgery. Among the 309 subjects, 144 (46.6%) of them exhibited hypertension before surgery, a significantly higher ratio than that of the whole population. One week after surgery, blood pressure of 106 (73.6%) patients turned back to normal. Blood pressure of another 37(25.7%) patients decreased with different degrees, although still higher than normal. Moreover, it appears that both approaches were effective in improving blood pressure, while the posterior approach was more effective in decreasing systolic blood pressure. We speculate this type of hypertension might result from hyperactivity of sympathetic nervous system as the heart rate of these patients decreased after surgery as well. Collectively, compression of spinal cord in CSM patients might be associated with hypertension, and decompression surgery largely attenuated this type of hypertension. These findings prove CSM to be a potential associated factor of high blood pressure and may shed light on therapies of hypertension in clinics.

## Introduction

Hypertension is a most prevalent disease in clinics, and as a chronic pathological condition, it can result in a variety of serious complications [1, 2]. Despite the undefined genetic and environmental influences, various factors are found to be associated with the pathogenesis of hypertension, which include cardiac and vascular remodeling, increased cardiac output and total peripheral resistance [3], diminished production or response to vasodilators, inflammation, immune reaction [4], abnormal cell signaling such as vascular and renal signaling [5], renal dysfunction [3], arterial baroreceptor adaptation, over activity of renin-angiotensin-aldosterone system (RAAS) [6, 7] and so on. With the development of techniques that allow direct or indirect quantification of adrenergic cardiovascular influences, there is increasing evidence that hyperactivity of sympathetic nervous system is involved in the pathogenesis of essential hypertension [8]. However, the mechanisms of hypertension are still under investigation.

Cervical spondylotic myelopathy (CSM) is a dominant non-traumatic type of myelopathy, caused by cervical intervertebral disc herniated into vertebral tube and squeezed spinal cord [9]. CSM can lead to a variety of signs and symptoms, and none of them is pathognomonic [9–11]. Severity of CSM can be evaluated through the Japanese Orthopaedic Associateion (JOA) scale, which involves motor function and sensory function of upper as well as lower extremities, trunk sensory function and bladder function. In addition, neck pain, headache, shoulder pain are also common symptoms. Upper motor neuron findings such as spasticity, hyperreflexia, Babinski’s sign may also be present [12, 13].

Treatment strategies for CSM depend on the severity of myelopathy, the extent of the disease process and a number of patient etiological factors [14]. It is well accepted that surgical management can effectively improve the symptoms and quality of life for patients who have experienced symptoms for an extended period of time, or who experienced disease progression [15, 16]. Surgery for CSM can be divided into anterior approach, posterior approach and the combination of the both. Anterior approach, also known as anterior cervical canal decompression approach, typically comprises corpectomy and anterior cervical discectomy and fusion (ACDF). Meanwhile, posterior approach typically comprises of laminectomy and laminoplasty. The selection of surgical strategy depends on a variety of factors, such as the cause of compression, the primary site of compression, the number of levels involved, the sagittal alignment of the spine and the clinical conditions of the patients [13, 17].

In this study, we found a large part of patients that suffered from CSM also exhibited hypertension, and drug intervention is sometimes required to control the high blood pressure. Intriguingly, blood pressures of these patients were decreased after the decompression surgery for CSM, and the posterior approaches seemed to be more effective in decreasing systolic blood pressure. Moreover, heart rate decreased along with the blood pressure as well. Taken together, we deduced this CSM associated hypertension may derive from hyperactivity of sympathetic nervous system. These results provided a potential index when selecting surgical strategy and a novel mechanism of increased sympathetic nervous activity.

## Materials and Methods

### Ethics statements

Given the retrospective nature of the study, written consent was not obtained. However, we got the oral consent from all participants in the study by telephone contact, and patient records were anonymized and de-identified prior to analysis. Then related data were extracted from hospital’s electronic and written medical records. The study was reviewed and obtained the approval from Institutional Review Board of Qilu Hospital, Shandong University.

### Subjects

From January 2011 to May 2014, three hundred and thirteen patients visited the authors’ department who met the following criteria were selected: (1) Patients of 18 years or older, had symptomatic CSM confirmed by CT and MRI imaging. As chronic pain attributes to hypertension [18], we chose patients with a visual analog scale (VAS) score under 3 to exclude pain as a factor of hypertension. (2) Patients were treated either by anterior or posterior decompressive/reconstructive approaches at the discretion of the operative teams. (3) Patients had complete medical records, including detailed records of blood pressure within one week. Blood pressure was measured on daily basis after operation, and we took the measurement data from the second day, so that anesthetics were metabolized completely and blood pressure data was objective. (4) Patients with obvious sympathetic symptoms such as vertigo and dizziness which implied sympathetic cervical spondylosis were excluded. (5) None of the patients developed iatrogenic spinal cord injury and the peri/postoperative course was uneventful in all. Of all 313 subjects, three of them received combined anterior and posterior approach, one patient was not followed-up properly. After excluding these four patients, 309 subjects were left for the research.

### Blood pressure measurement and grading

The blood pressure data are presented as average early morning values within a week before and after surgery. Blood pressure data on the day of surgery was excluded as anesthesia may decrease blood pressure. The blood pressure measurements were determined by the average results of 2 readings of systolic and diastolic blood pressure obtained at five minutes intervals using an electronic sphygmomanometer by trained nurses. A third measurement was performed when there was a difference more than 5mmHg between the two measurements. Blood pressure was assessed after five minutes of rest in the sitting position using appropriate size cuffs before taking any antihypertensive drugs. According to the Sixth Report of the Joint National Committee (JNC) [19], which conforms to Chinese hypertension grading standard, hypertension can be graded according to the blood pressure level and divided into three stages: stage 1 hypertension, with systolic blood pressure (SBP) ranging from 140mmHg to 159mmHg, diastolic blood pressure (DBP) ranges from 90mmHg to 99mmHg; stage 2 hypertension, with SBP ranging from 160mmHg to 179mmHg, DBP ranges from 100mmHg to 109mmHg; stage 3 hypertension, with SBP greater than 180mmHg or DBP greater than 110mmHg. Blood pressure of patients should be classified into the higher stage when SBP and DBP belong to different stages. The JNC 7 report introduced a new term “prehypertension” for those with BPs ranging from 120 to 139 mmHg systolic and/or 80 to 90 mmHg diastolic blood pressure [20], so we also analyzed blood pressure of patients that belonged to the range.

### Heart rate measurement

The heart rate data are presented as average early morning values within a week before and after surgery. As the electronic sphygmomanometer that measuring blood pressure records the current heart rate as well, the heart rate measurements were determined by the average results of 2 readings, recorded along with blood pressure.

### Statistical analysis

Data are expressed as mean value and the standard deviation (SD). Paired t-test was used for the analysis of the blood pressures before and after surgery. For the comparison of anterior and posterior approaches, unpaired t-test was used. Chi-square test was used to analyze the proportion among different hypertension stages. P-values<0.05 were considered to be statistically significant. All statistical analyses were conducted using SAS version 9.2 (SAS Institute Inc, Cary, NC).

## Results

### Ratio of hypertension was significantly higher in CSM patients compared with controls

Of the 309 subjects, 144 patients were diagnosed with hypertension before surgery according to values of their blood pressure, which means that 46.6% of our CSM patients were combined with different degrees of hypertension. It indicated the ratio of hypertension was significantly elevated in CSM patients compared with the average prevalence of the same age group in the same region as reported [21].

### Both anterior and posterior approaches of surgery were effective for attenuation of hypertension in CSM patients

**Fig 1:**
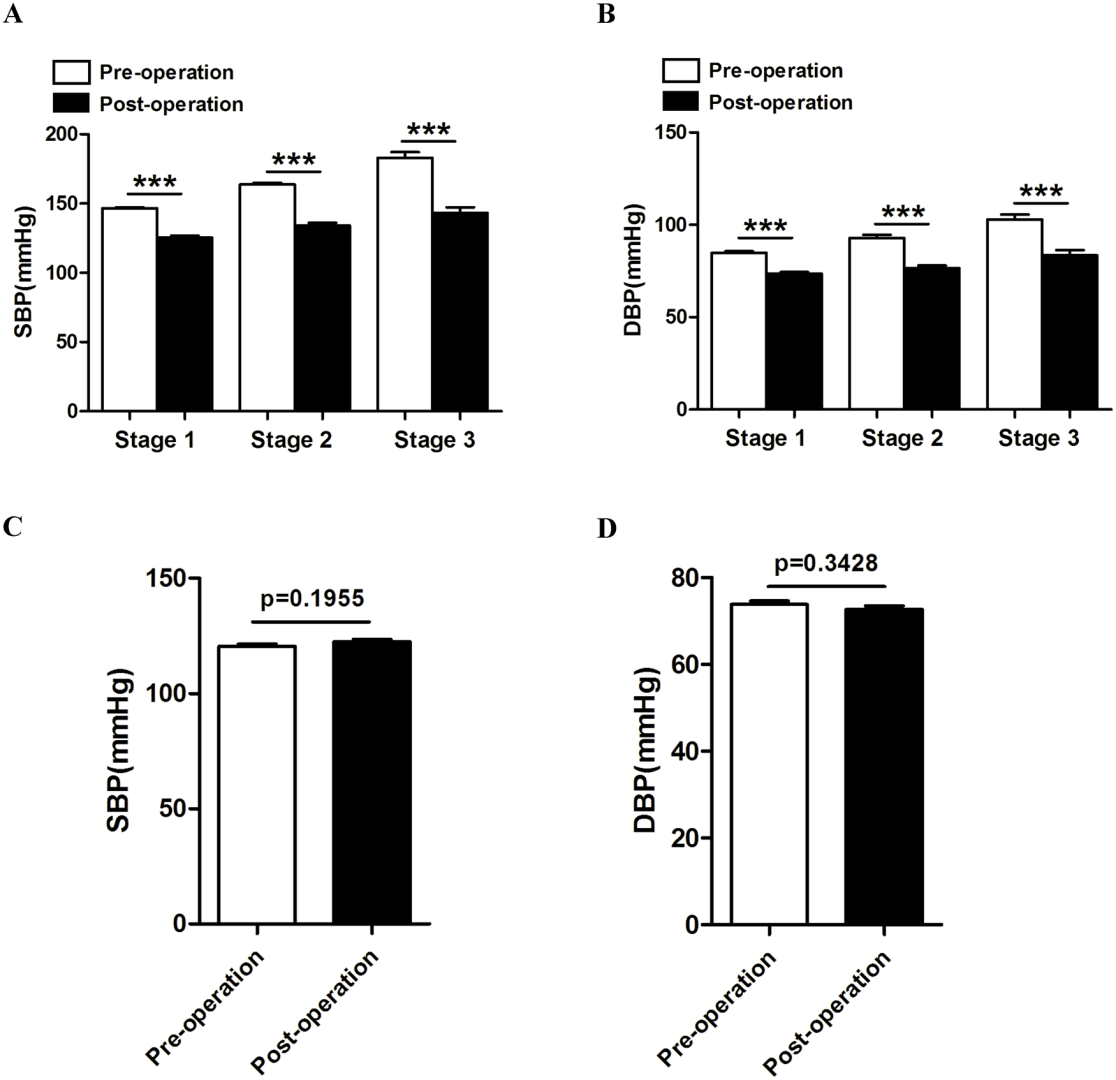
Changes of blood pressure after surgery for CSM. (A) Surgery for CSM decreased SBP significantly in three hypertension stages. (B) Surgery for CSM decreased DBP significantly in three hypertension stages. ***p<0.001. (C)Surgery for CSM had no effect on SBP in normotensive patients. (D) Surgery for CSM had no effect on DBP in normotensive patients.

As is shown in Fig 1A and 1B, both anterior and posterior approaches were effective for decreasing blood pressure of CSM patients accompanied with hypertension. The average blood pressure of these patients decreased from 155.5/89.1mmHg to 131.8/76.6mmHg after surgery for CSM. Among the 144 patients who were diagnosed with hypertension before decompression surgery, 87(60.4%) had stage 1 hypertension, 40(27.8%) had stage 2 hypertension and 17(11.8%) had stage 3 hypertension. After the surgery, the average blood pressure of stage 1 hypertension decreased from 146.4/84.6mmHg to 127.1/74.6mmHg, among the 87 patients, 71(81.6%) patients had their blood pressures restored to normal, 15(17.2%) patients were still in the range of stage 1 hypertension. There was a patient whose blood pressure increased abnormally, which might be variation among individuals. The average blood pressure of stage 2 hypertension decreased from 163.8/92.9mmHg to 134.0/76.4mmHg after the surgery. Blood pressures of 28(70.0%) patients were back to normal, 9(22.5%) patients had downgraded to stage 1 hypertension, and blood pressures of 3(7.5%) patients decreased but were still in stage 2 hypertension. In case of stage 3 hypertension, the average blood pressure decreased from 182.9/102.9mmHg to 142.2/83.5mmHg after the surgery. 7(41.2%) patients’ blood pressure returned to normal range, 8(47.0%) patients were degraded to stage 1 hypertension and 2(11.8%) patients were degraded to stage 2 hypertension. Statistical analysis indicated that operation for the treatment of CSM may decrease the blood pressure of stage 1, 2 and 3 hypertension patients effectively. (p<0.0001) However, surgery for CSM had no effect on either SBP or DBP in normotensive patients. (Fig 1C and 1D)

Table 1 provides a summary of the surgical outcome. The ratio of hypertensive patients of all the three stages decreased significantly after the surgery. No significant difference was shown in VAS before and after surgery, indicating that pain is not involved in the pathogenesis of hypertension here.

**Table 1:** Surgical Outcomes.

	Pre-operation	Post-operation	P Value
**VAS**	**2.1±0.6**	**2.0±0.6**	**0.4533**
**Patients with hypertension**	**46.60%(144/309)**	**12.30% (38/309)**	**< 0.0001**
**Stage 1 hypertension**	**28.16%** (**87/309)**	**10.36% (32/309)**	**< 0.0001**
**Stage 2 hypertension**	**12.95%(40/309)**	**1.94% (6/309)**	**< 0.0001**
**Stage 3 hypertension**	**5.50%(17/309)**	**0 (0/309)**	**< 0.0001**

VAS data are given as mean±SD.

VAS, visual analog scale.

In addition, there were another 14 of the 309 patients that belong to prehypertension state, and surgery for the treatment of CSM diminished the blood pressure of these patients from 131.2/76.6mmHg to 117.8/67.9mmHg, which made all these patients become real normotensive.

Table 2 summarizes the analysis of changes in the outcomes parameters in accordance to anterior and posterior approaches to treat CSM. We observed that subjects in the anterior group were younger (52.2 vs. 58.2; p< 0.0001) No significant differences were shown in gender structure (p = 0.4568) and BMI (0.3656) between the two groups. Other confounding factors such as alcohol drinking, smoking, chronic disease including diabetes and coronary heart disease didn’t affect surgical strategy. The ratio of patients that are complicated by hypertension was of no significant difference between the two approaches (p = 0.1279). For the ratio of three stages of hypertension, no significant difference was observed. There were 8 subjects of stage 3 hypertension treated by the anterior approach. After surgery, blood pressure of 2 (25%) subjects was back to normal, 5 (62.5%) subjects’ blood pressure improved to stage 1 hypertension and 1 (12.5%) subject belonged to stage 2 hypertension. By contrast, 9 subjects of stage 3 hypertension were treated by posterior approach, which resulted in 5 (55.6%) subjects back to normal, 3 (33.3%) subjects assigned to stage 1 hypertension and 1 (11.1%) subject to stage 2 hypertension. Besides, 20 subjects of stage 2 hypertension were in the anterior approach group, which resulted in 12 (60.0%) subjects back to normal, 5 (25.0%) subjects back to stage 1 hypertension and 3 (15.0%) subjects were still in stage 2 hypertension. On the contrary, the posterior approach turned 16 (80.0%) of 20 subjects of stage 2 hypertension to normal, 4 (20.0%) subjects to stage 1 hypertension. Additionally, there were 53 stage 1 hypertension subjects who underwent anterior approach surgery, in which 42 (79.2%) subjects were back to normal, 10 other subjects still belonged to stage 1 hypertension. The posterior approach resulted in 29 (85.3%) of 34 stage 1 hypertensive patients become normotensive, 5 (14.7%) patients were still of the same stage as pre-operation.

**Table 2:** Baseline Characteristics of Subjects (N = 309).

	Anterior(N = 189)	Posterior(N = 120)	P Value
**Age(yr)**	**52.2±9.6**	**58.2±10.1**	**< 0.0001**
**No.(male: female)**	**189(124/65)**	**120(84/36)**	**0.4568**
**VAS**	**2.0±0.5**	**2.1±0.6**	**0.5116**
**BMI**	**23.7±3.2**	**24.1±2.8**	**0.3656**
**Patients that drinking**	**26.46%(50/189)**	**27.5%(33/120)**	**0.8954**
**Smokers**	**23.81%(45/189)**	**25%(30/120)**	**0.8918**
**Patients with diabetes**	**6.35%(12/189)**	**7.5%(9/120)**	**0.8172**
**Patients with coronary heart disease**	**5.29%(10/189)**	**5.83%(7/120)**	**1.0000**
**Patients with hypertension**	**42.86%(81/189)**	**52.50%(63/120)**	**0.1279**
**Stage 1 hypertension**	**65.43%** (**53/81)**	**53.97% (34/63)**	**0.1739**
**Stage 2 hypertension**	**24.69% (20/81)**	**31.75% (20/63)**	**0.3563**
**Stage 3 hypertension**	**9.88% (8/81)**	**14.28% (9/63)**	**0.4452**

Data of age, VAS and BMI are given as mean±SD.

VAS, visual analog scale; BMI, body mass index.

### Posterior approach exhibited better outcome for SBP, while no dramatic difference was observed in diminishing DBP between two approaches

As is shown in Table 3, the average SBP of stage 1 hypertension decreased from 147.1 mmHg to 126.5 mmHg after the posterior approach surgery, which was significantly effective than the anterior approach(The anterior approach decreased the blood pressure of stage 1 hypertensive subjects from 146.0 mmHg to 129.2 mmHg; p = 0.0270). As for stage 2 hypertension, the posterior approach presented a better effect by decreasing subjects’ blood pressure from 164.4 mmHg to 131.8 mmHg, while the average blood pressure of anterior group was 163.1 mmHg to 138.0 mmHg (p = 0.0221). The posterior group decreased the blood pressure of stage 3 hypertensive subjects more effectively than the anterior groups as well (From 183.8 mmHg to 142.3 mmHg versus 182.0 mmHg to 142.1 mmHg; p = 0.0109). No significant difference was shown between the two groups when it came to DBP. The posterior approach decreased blood pressure of 3 different stages from 82.6 mmHg to 73.6 mmHg, 92.2 mmHg to 74.3 mmHg and 99.9 to 80.7 mmHg respectively. While the anterior approach showed a similar effect, by separately decreasing blood pressure of 3 stages from 86.1 mmHg to 76.0 mmHg (p = 0.9389), 93.4 mmHg to 80.0 mmHg (p = 0.4419) and 106.3 mmHg to 86.6 mmHg (p = 0.9599).

**Table 3:** Different Changes of Subjects’ Blood Pressure Classified by Surgical Approach (N = 309).

	Hypertension Grade	Anterior(N = 189)		Posterior(N = 120)		
		Pre-operation	Post-operation	Pre-operation	Post-operation	P Value
	**Stage 1**	**146.0±5.9**	**129.2±14.0**	**147.1±6.5**	**126.5±14.1**	**0.0270**
**SBP(mmHg)**	**Stage 2**	**163.1±6.6**	**138.0±16.8**	**164.4±8.3**	**131.8±10.1**	**0.0221**
	**Stage 3**	**182.0±10.5**	**142.1±12.3**	**183.8±21.5**	**142.3±14.8**	**0.0109**
	**Stage 1**	**86.1±6.9**	**76.0±8.2**	**82.6±8.2**	**73.6±11.6**	**0.9389**
**DBP(mmHg)**	**Stage 2**	**93.4±10.0**	**80.0±10.6**	**92.2±11.1**	**74.3±7.9**	**0.4419**
	**Stage 3**	**106.3±7.5**	**86.6±8.4**	**99.9±13.2**	**80.7±13.7**	**0.9599**

Data are given as mean±SD.

SBP, systolic blood pressure; DBP, diastolic blood pressure.

**Fig 2:**
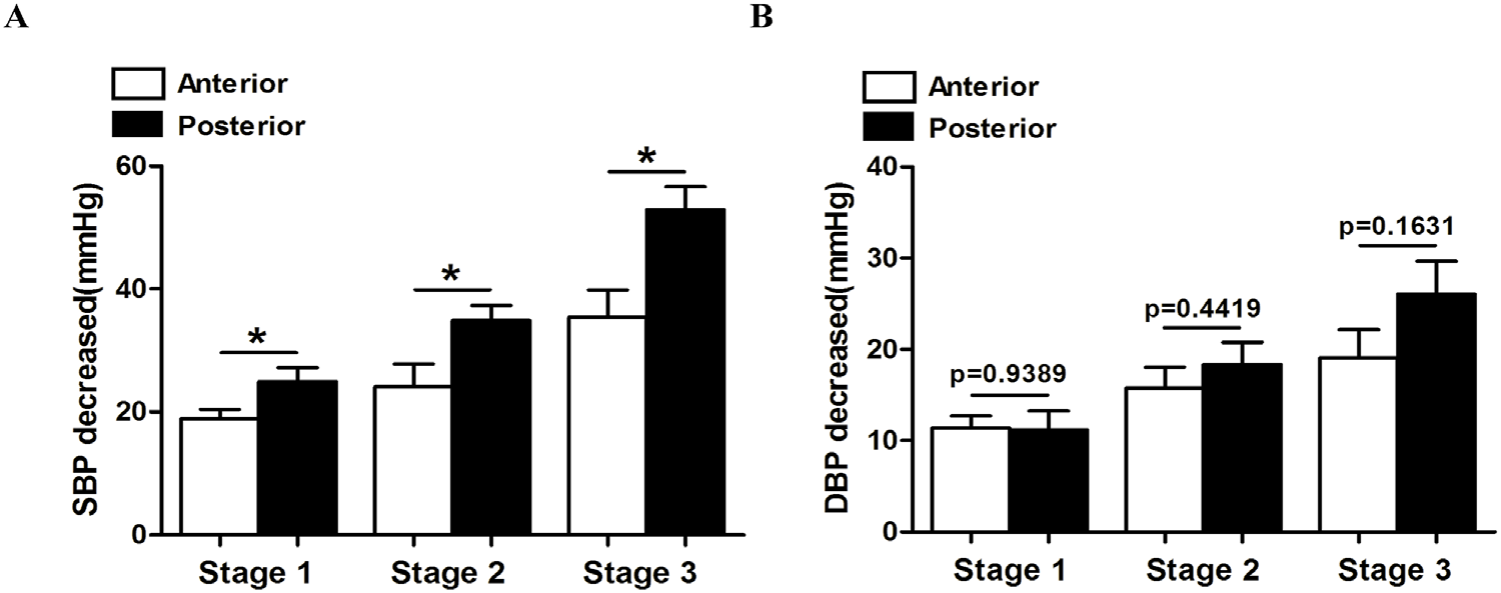
Comparison of the two approaches in decreasing blood pressure. (A) Changes of SBP after surgery was analyzed to investigate which approach is more effective. (B) Changes of DBP after surgery was analyzed to investigate which approach is more effective. *p<0.05.

As shown in Fig 2, therapeutic effect of the two surgical approaches in decreasing hypertensive patients’ blood pressure were summarized, and the result implied that the posterior approach may exhibits better outcome in SBP (Fig 2A) but not in DBP (Fig 2B).

### Surgery for the treatment of CSM could decrease heart rate of hypertensive patients

**Fig 3:**
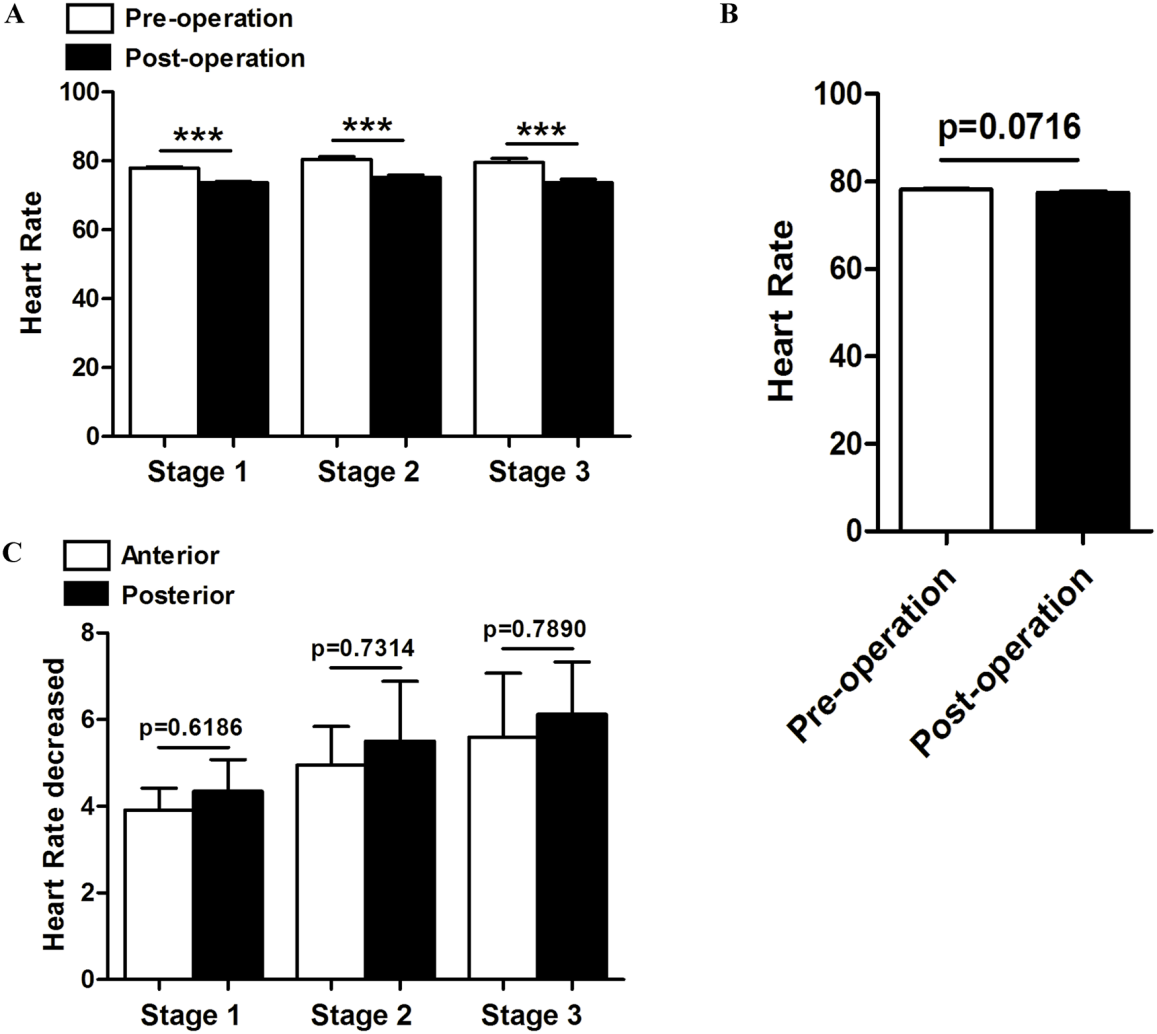
Changes of heart rate after surgery for CSM. (A) Surgery for CSM decreased heart rate significantly in three hypertension stages. (B) Changes of heart rate after surgery in normotensive patients. (C) Comparison of the two approaches in decreasing heart rate in hypertensive patients. ***p<0.001.

From the perspective of anatomical positional relationship, we speculate CSM associated hypertension may results from the irritation of sympathetic nervous system. To confirm our speculation, we analyzed the changes of heart rate after surgery, as heart rate could represent sympathetic nerve activity to some extent. Consistent with the changes of blood pressure, surgery for CSM significantly decreased heart rate of hypertensive patients. (Fig 3A) Meanwhile, in patients with normal blood pressure, these surgeries didn’t affect their heart rate. (Fig 3B) In addition, there is no significant difference between the two approaches in decreasing heart rate. (Fig 3C)

## Discussion

To date, the prevailing treatment of hypertension is pharmacologic therapy and a large number of drugs are currently available for improving blood pressure. In many cases, drug combinations are required, as more than two-thirds of patients with hypertension cannot be controlled on one type of such drugs. Moreover, most of them face a lifetime medication in order to avoid the severe complications of high blood pressure. Therefore, the etiology and relevant therapy remains to be further investigated. Here we reported a potential etiology of hypertension—CSM associated hypertension, a kind of hypertension that might be cured by decompression surgery.

This retrospective observation and statistic work demonstrates that a large part of CSM patients also suffered from hypertension. Moreover, both anterior approach and posterior approach decompression surgeries decreased blood pressure of these patients to a certain degree. What’s more, surgeries had no effect on blood pressure in normotensive patients. Hypertension in these patients were improved or even cured after the surgery, and the blood pressure of some patients with uncontrolled hypertension became stable and controlled following the decompression operation. Furthermore, according to our findings of this study, the posterior group appeared to be more effective in decreasing SBP. CSM is known to be the most common cause of acquired neurological disability in those older than 50 years [22]. Both CSM and hypertension are chronic progressive diseases that are associated with aging, and our findings from the current study implied a potential relationship between CSM and hypertension. 46.6% of our subjects with CSM also suffered from hypertension, which was significantly more than the average prevalence of hypertension in the same region [23, 24]. We termed this type of hypertension “CSM associated hypertension”, as it seemed that a positive relationship exists between CSM and hypertension. The renocentric view of blood pressure regulation has predominated for the last 30–40 years [25–27]. Recently, some reports have demonstrated that the sympathetic nervous system plays a critical role in the pathogenesis of hypertension [28, 29]. In consideration of the anatomical positional relationship, hyperactivity of the sympathetic nervous system may serve as a possible explanation for the genesis of “CSM associated hypertension”. The changes of heart rate after surgery further supported our deduction as resting heart rate is one of the most important indirect hemodynamic markers of the adrenergic function [8]. The main symptoms of the subjects here were typical CSM symptoms, and subjects’ VAS scores were under 3. Moreover, no significant difference in VAS was shown after surgery, which excluded chronic pain as the probable reason for hypertension in this study. In addition, pre-operative anxiety may also leads to transient blood pressure increase. However, it usually happens hours before surgery [30, 31]. A possible explanation of this type of hypertension is the increase of sympathetic activity resulted from the chronic irritation and compression of the vertebral canal. It is well known that cervical spinal tissues are rich in sympathetic fibers and the cervical sympathetic trunk consists of a main trunk and 2–4 ganglia that are located anterior to the transverse processes [32, 33]. In the process of CSM mediated hypertension, herniated intervertebral discs and spinal stenosis may be the initiating factors, followed by the increased activation of sympathetic nervous system and constriction of blood vessels. More importantly, the cervical dura mater and the posterior longitudinal ligament have distinct sympathetic innervation patterns [34]. Irritating of the spinal cord, dura mater or longitudinal ligament activated sympathetic nervous system, resulting in relevant feedback to limit the blood pressure-raising effects of high sympathetic activity [28]. However, aging is associated with oxidative stress, which may suppress this feedback through affecting bioavailability or the production of nitric oxide, an important molecule that could induce vasodilation, especially in humans over 40 years old [35–38]. As mentioned above, the average age of our subjects was 54.6 and 96.5% of the subjects were older than 40. Collectively, the increased activity of sympathetic nervous system caused by CSM may be an explanation for hypertension.

It is reported that anterior and posterior surgical approaches have equivalent efficacy in treating CSM [39]. Here we present blood pressure as a potential outcome parameter. This parameter may give a hint to the choice of surgical strategy. Generally, the surgical approaches elected are dependent upon multiple factors, such as the cause of compression, the site of compression, the number of levels involved, patient age and the surgeon’s familiarity [17, 39]. Our observation implies blood pressure as another factor to be taken into consideration. In line with the previous study, our patients of the posterior group were older [39]. Both approaches could improve patients’ hypertensive condition, while it appears that the posterior strategy is slightly more effective, as the posterior approach decreased SBP of these patients by a greater degree, which is a major risk factor for hypertension.

Our study has a number of limitations that warrant mention. Given the retrospective nature of this study, more detailed data that could reflect the sympathetic nerve activity such as norepinephrine and MSNA are lacked. Besides, other compounding factors that may contribute to the pathogenesis of hypertension are not put into this study. In addition, the long-term effect of decompression surgery on blood pressure requires further observation. To aid the evidence of CSM associated hypertension, whether hypertension develops following the occurrence of CSM deserves to be further investigated. Animal experiments as well as prospective randomized controlled clinical trials are warranted in order to figure out the pathogenesis and mechanism of CSM associated hypertension.
